# Patterns and correlates of mental healthcare utilization during the COVID-19 pandemic among individuals with pre-existing mental disorder

**DOI:** 10.1371/journal.pone.0303079

**Published:** 2024-06-04

**Authors:** Hyunjoon Lee, Chris J. Kennedy, Allison Tu, Juliana Restivo, Cindy H. Liu, John A. Naslund, Vikram Patel, Karmel W. Choi, Jordan W. Smoller

**Affiliations:** 1 Psychiatric and Neurodevelopmental Genetics Unit, Center for Genomic Medicine, Massachusetts General Hospital, Boston, Massachusetts, United States of America; 2 Department of Psychiatry, Center for Precision Psychiatry, Massachusetts General Hospital, Boston, Massachusetts, United States of America; 3 Department of Biomedical Informatics, Vanderbilt University Medical Center, Nashville, Tennessee, United States of America; 4 Department of Psychiatry, Massachusetts General Hospital, Boston, Massachusetts, United States of America; 5 Harvard College, Cambridge, Massachusetts, United States of America; 6 Department of Global Health and Social Medicine, Harvard Medical School, Boston, Massachusetts, United States of America; 7 Departments of Pediatrics and Psychiatry, Brigham and Women’s Hospital, Boston, Massachusetts, United States of America; 8 Harvard Medical School, Boston, Massachusetts, United States of America; 9 Department of Global Health and Population, Harvard T.H. Chan School of Public Health, Boston, Massachusetts, United States of America; 10 Harvard T.H. Chan School of Public Health, Boston, Massachusetts, United States of America; Murdoch University, AUSTRALIA

## Abstract

How did mental healthcare utilization change during the COVID-19 pandemic period among individuals with pre-existing mental disorder? Understanding utilization patterns of these at-risk individuals and identifying those most likely to exhibit increased utilization could improve patient stratification and efficient delivery of mental health services. This study leveraged large-scale electronic health record (EHR) data to describe mental healthcare utilization patterns among individuals with pre-existing mental disorder before and during the COVID-19 pandemic and identify correlates of high mental healthcare utilization. Using EHR data from a large healthcare system in Massachusetts, we identified three “pre-existing mental disorder” groups (PMD) based on having a documented mental disorder diagnosis within the 6 months prior to the March 2020 lockdown, related to: (1) stress-related disorders (e.g., depression, anxiety) (N = 115,849), (2) serious mental illness (e.g., schizophrenia, bipolar disorders) (N = 11,530), or (3) compulsive behavior disorders (e.g., eating disorder, OCD) (N = 5,893). We also identified a “historical comparison” group (HC) for each PMD (N = 113,604, 11,758, and 5,387, respectively) from the previous year (2019). We assessed the monthly number of mental healthcare visits from March 13 to December 31 for PMDs in 2020 and HCs in 2019. Phenome-wide association analyses (PheWAS) were used to identify clinical correlates of high mental healthcare utilization. We found the overall number of mental healthcare visits per patient during the pandemic period in 2020 was 10–12% higher than in 2019. The majority of increased visits was driven by a subset of high mental healthcare utilizers (top decile). PheWAS results indicated that correlates of high utilization (prior mental disorders, chronic pain, insomnia, viral hepatitis C, etc.) were largely similar before and during the pandemic, though several conditions (e.g., back pain) were associated with high utilization only during the pandemic. Limitations included that we were not able to examine other risk factors previously shown to influence mental health during the pandemic (e.g., social support, discrimination) due to lack of social determinants of health information in EHR data. Mental healthcare utilization among patients with pre-existing mental disorder increased overall during the pandemic, likely due to expanded access to telemedicine. Given that clinical correlates of high mental healthcare utilization in a major hospital system were largely similar before and during the COVID-19 pandemic, resource stratification based on known risk factor profiles may aid hospitals in responding to heightened mental healthcare needs during a pandemic.

## Introduction

The COVID-19 pandemic has represented a major societal stressor with substantial impacts on mental health morbidity across the entire population [1–4]. Individuals with pre-existing mental disorders may have been particularly vulnerable to mental health difficulties during the pandemic. For example, studies have found that those with pre-existing mental disorder experienced more psychiatric symptoms such as anxiety, depression, stress, and sleep problems during the pandemic [5–11]. However, it is unclear how this increased burden of psychiatric symptoms among individuals with pre-existing mental disorder have translated to mental healthcare utilization. Characterizing changes in mental healthcare utilization during the pandemic may inform health system efforts to prepare for and adapt to future pandemics or similar crises.

Electronic health record (EHR) data provide a particularly valuable resource for characterizing mental healthcare utilization since they capture all mental health encounters (e.g., hospital visits, medical procedures, etc.) in a healthcare system, both in-person and telemedicine. Prior studies using EHR data to examine mental healthcare utilization during the COVID-19 pandemic found that outpatient mental health visit volume during the pandemic exceeded pre-pandemic levels [12, 13], driven by the uptake of telemedicine visits; however, these studies did not focus on patients with pre-existing mental disorder who may represent a particularly at-risk group. A register-based study from Italy reported reduced psychiatric hospitalizations among patients with pre-existing mental disorder in the first two months of the pandemic but did not examine broader mental health services (e.g., outpatient) and longer term outcomes [14]. Thus, changes in overall mental healthcare utilization among people with pre-existing mental disorder during the pandemic period remain unclear. Understanding utilization patterns of these at-risk individuals and identifying those most likely to exhibit increased utilization could improve patient stratification and efficient delivery of mental health services.

Here, we used EHR data from Mass General Brigham (MGB), the largest healthcare system in Massachusetts, to examine temporal variation in mental healthcare utilization among patients with pre-existing mental disorders during the early phase of the pandemic (March—December 2020), and to identify clinical correlates that might underpin high mental healthcare utilization. Specifically, we sought to (1) characterize monthly variation in mental healthcare visits during the first nine months of the pandemic and (2) identify clinical history correlates of high mental healthcare utilization. We expected to observe differences in mental healthcare utilization before and during the pandemic, specifically an initial decrease in utilization during the acute lockdown period (March—May 2020). As for correlates of high mental healthcare utilization, we hypothesized that some clinical conditions correlates (e.g., those posing a risk for severe COVID-19 outcomes, e.g., heart diseases, lung diseases, cancer, diabetes) might differentiate those with high mental healthcare utilization.

## Materials and methods

### Ethics statement

This study adhered to the principles outlined in the Declaration of Helsinki and International Conference on Harmonization Good Clinical Practice guidelines. This study was approved by the MGB Human Research Committee (MGBHRC), the Institutional Review Board (IRB) of MGB (#2020P003089), and informed consent was waived by the IRB.

### Data source

Our data source was the MGB Research Patient Data Registry (RPDR) [15]. The MGB RPDR is a data warehouse that covers over 20 years of EHR data from over 6.5 million patients seen in Massachusetts General Hospital, Brigham and Women’s Hospital, and other affiliated community and specialty hospitals in the Boston area. Secondary analysis of these de-identified data was approved by the MGB Institutional Review Board. Our EHR data was de-identified to exclude Health Insurance Portability and Accountability Act (HIPAA) identifiers including names, addresses, medical record numbers, among others. While dates are also considered HIPAA identifiers and necessary for our longitudinal analyses, all dates were shifted by a random (small) number of days for each patient to preserve anonymity. The patient population eligible for this study was restricted to patients with at least one clinical note and three visits since 2005 with each visit more than 30 days apart as a data floor for identifying patients active in the system over a period of time (N = 2,919,242).

### Group definitions

We defined three pre-existing mental disorder groups (PMD) based on having a documented mental disorder diagnosis within the 6 months prior to the public health emergency declaration on 3/13/2020 (9/14/2019–3/12/2020): (1) stress-related disorders group (major depressive disorder (MDD), anxiety disorder, post-traumatic stress disorder (PTSD), adjustment reaction, acute reaction to stress) (N = 115,849) (2) serious mental illness group (schizophrenia, psychosis, bipolar disorder) (N = 11,530), or (3) compulsive behavior disorders group (eating disorder, obsessive compulsive disorder (OCD), body dysmorphic disorder) (N = 5,893). The patient groups were conceptually defined based on distinct categories of conditions anticipated to manifest heightened exacerbation or vulnerability amidst the pandemic: stress-related disorders, owing to their stress-associated nature; serious mental illnesses, due to their severity; and disorders characterized by compulsive behaviors, potentially triggered by aspects of illness or infection. Group membership was determined by having at least one relevant PheWAS code (phecode; a grouping of International Classification of Diseases (ICD, ninth and tenth edition)) [16]. The full list of phecodes pertaining to each PMD is provided in S1 Table. We aimed to capture those with a relatively recent mental healthcare diagnosis (prior 6 months) rather than those less likely to have active mental health conditions or who had left the health system. Individuals could belong to multiple PMD groups as long as they had the relevant phecodes; thus, resulting groups are not mutually exclusive but rather reflect individuals who were recently seen in the hospital system for each given mental disorder category (see S1 Fig).

**Fig 1:**
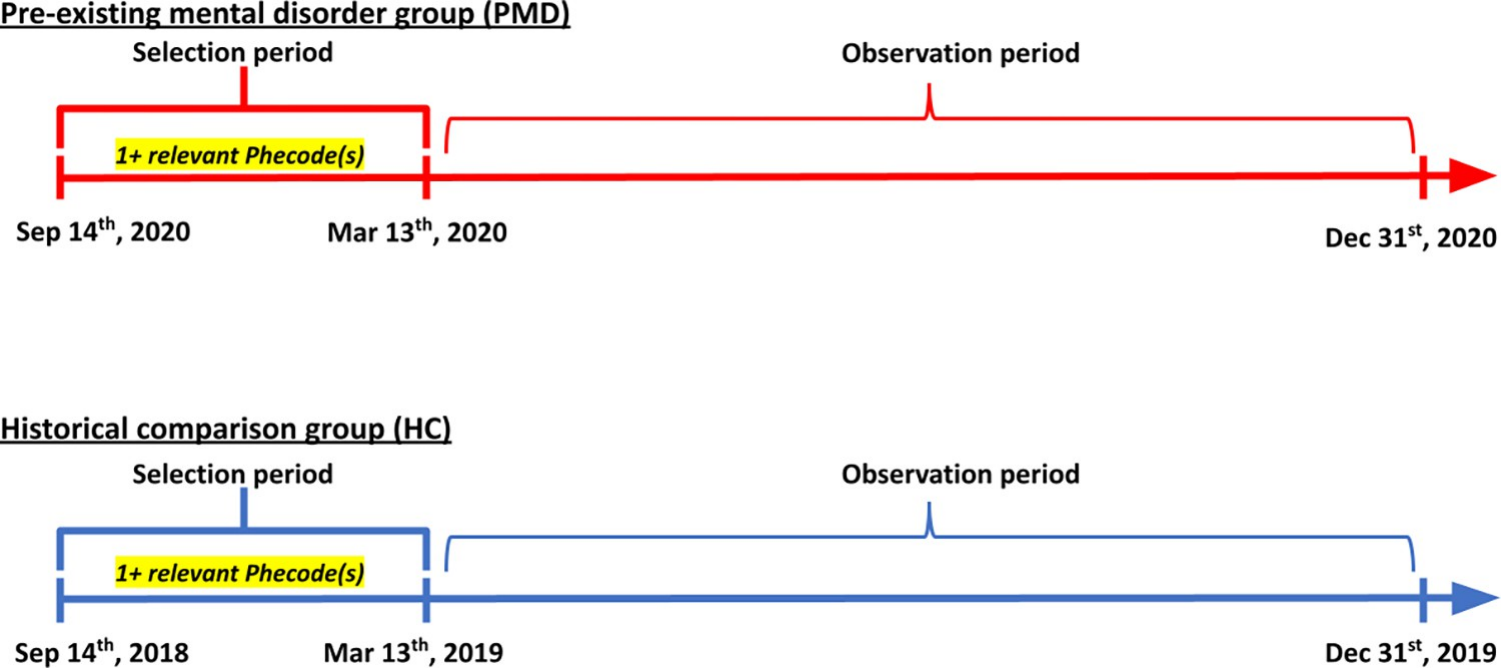
Patient selection process for pre-existing mental disorder group (PMD) and historical comparison group (HC).

For each PMD (stress-related disorders, serious mental illness, and compulsive behavior disorders), we identified a “historical comparison” group (HC) (N = 113,604, 11,758, and 5,387, respectively) using the same criteria of having at least one relevant phecode during the 6 months prior to March 13 during the previous year (9/14/2018–3/12/2019). A graphical description of patient selection process is provided in Fig 1.

### Descriptive patterns of mental healthcare visits

First, we sought to examine the monthly number of mental healthcare visits for PMDs in 2020 and HCs in 2019. We defined mental healthcare visits as unique EHR-documented visit dates with an associated ICD-10 F code (mental, behavioral, and neurodevelopmental disorders). Our analysis focused on mental healthcare visits during the first nine months of post-lockdown period (3/13/2020–12/31/2020 for PMDs; 3/13/2019–12/31/2019 for HCs). We selected the first nine months to ensure that HCs’ mental healthcare visits were not counted during a period in which COVID-19 entered the conversation in the United States, which was January 2020.

Next, we compared the mean total and quarterly number of per-patient mental healthcare visits of the PMDs and HCs during the first nine months of the post-lockdown period. We used Welch’s t-test to examine whether the per-patient mental healthcare visits were significantly different between the PMDs and HCs each month and quarter.

### High mental healthcare utilizers

Because we observed that PMDs had a higher mean total number of per-patient mental healthcare visits than the HCs, we investigated whether this difference was due to a uniform increase in the distribution of mental healthcare visits across the study population or driven by a subset of high mental healthcare utilizers. We qualitatively compared the proportion of mental healthcare visits accounted for by each mental healthcare utilization percentile in PMDs and HCs. Then, to identify individuals who contributed to the excess visits during the pandemic, we calculated the ratio of the proportion of mental healthcare visits accounted for by each percentile in PMDs to that of the HCs and identified the percentiles in which the ratio was greater than one (reflecting more visits during the pandemic). We used a per-percentile comparison because the sample sizes of the PMDs and the HCs were different.

Because the analysis described above showed that approximately the top decile of mental healthcare utilizers in each PMD was responsible for the excess visits during the pandemic, we defined high mental healthcare utilizers as individuals accounting for the top decile of mental health visits during the first nine months of the pandemic (3/13/2020–12/31/2020 for PMDs; 3/13/2019–12/31/2019 for HCs), similar to existing research [17]. We examined the differences in sociodemographic characteristics (sex, age, race, ethnicity, insurance type, non-mental healthcare visit count, and mental healthcare visit count) between the top decile and bottom nine deciles of mental health utilizers for both PMDs and HCs. We also compared the differences in healthcare utilization (non-mental healthcare visit count and mental healthcare visit count) between the top deciles of PMDs and HCs, to examine whether high mental healthcare utilizers during the pandemic were simply more ill in general and required care for a wide range of health concerns, including mental health. We performed Pearson’s chi-squared test to compare sex, race, ethnicity, and insurance type and Welch’s t-tests to compare age, non-mental healthcare visit count, and mental healthcare visit count across high utilizers from the PMDs versus HCs.

### Correlates of high mental healthcare utilization before and during the COVID-19 pandemic

We sought to identify and compare the clinical history correlates for high mental healthcare utilization (top decile) between PMD and HC for each group (stress-related disorders, serious mental illness, compulsive behavior disorders) using a PheWAS analysis [16]. First, we constructed the clinical history phenome by extracting all diagnoses for each PMD group via ICD-9 and ICD-10 codes and mapping them to phecodes using the *PheWAS* R package, time-censored to the past year at least one month prior to the lockdown (2/13/2019–2/13/2020) to establish temporal precedence [18]. Using logistic regression models, we estimated the association between the high mental healthcare utilization (defined as top decile vs. bottom nine deciles of utilization) and each clinical history phenotype (coded as present/absent based on having/not having at least two ICD codes), adjusting for sex, age, self-reported race, self-reported ethnicity, insurance type, and healthcare utilization (measured by total visit count during 2/13/2019–2/13/2020). Associations were computed only for phecodes with a minimum prevalence of 1% during the past year (2/13/2019–2/13/2020) to remove rare conditions. We removed the phecodes pertaining to each category (stress-related disorders, serious mental illness, compulsive behavior disorders; as shown in S1 Table) during the past year from the analysis. Also, to eliminate redundancy, we removed the three-digit parent phecodes (e.g., 300—anxiety disorders) if they had any child phecode with digits following the decimal point (e.g., 300.11—generalized anxiety disorder). For the HCs, the phecodes were time-censored to those during the same time period as that of the PMDs but for the previous year (2/13/2018–2/13/2019). Phecode associations below the Bonferroni-adjusted p-value (stress-related disorders group: 2.813e-05 for PMD and 2.806e-05 for HC; serious mental illness group: 2.907e-05 for PMD and 2.921e-05 for HC; compulsive behavior disorders group: 3.051e-05 for PMD and 3.079e-05 for HC) were considered statistically significant. While there were sizeable patient overlaps between the top deciles of PMDs and HCs (stress-related disorders: 46%, serious mental illness: 44%, compulsive behavior disorders: 36%), we avoided overlap between clinical history used for identifying risk factors for high utilization in 2019 and that of 2020 by implementing time-censorship, limiting the data spanning from February 13th, 2018 to 2019 for PheWAS related to high utilization in 2019 and from February 13th, 2019 to 2020 for PheWAS pertaining to high utilization in 2020. Lastly, we compared significant phecodes and their adjusted odds ratios between the PMD and HC groups by examining the p-value of the interaction coefficient between high mental healthcare utilization status and PMD versus HC membership, allowing us to identify phecodes that differed significantly between PMD and HC high utilizers.

## Results

### Sample characteristics

The sociodemographic characteristics of PMDs and HCs are shown in Table 1. Across both groups in each category (stress-related disorders, serious mental illness, compulsive behavior disorders), the majority of patients were female (55–69%) and self-identified as White (77–84%) and non-Hispanic (96–98%).

**Table 1:** Sociodemographic characteristics of PMDs in 2020 and HCs in 2019.

	Stress-related disorders group		Serious mental illness group		Compulsive behavior disorders group	
	**2020**	**2019**	**2020**	**2019**	**2020**	**2019**
**N**	115,849	113,604	11,530	11,758	5,893	5,387
**Sex**						
**Female**	68.4%	68.9%	54.8%	55.0%	68.4%	67.8%
**Male**	31.6%	31.1%	45.2%	45.0%	31.6%	32.2%
**Race**						
**White**	81.5%	81.2%	77.2%	77.3%	82.2%	83.5%
**Black**	5.0%	5.3%	9.4%	9.6%	5.4%	4.1%
**Asian**	2.6%	2.5%	2.5%	2.5%	2.5%	2.7%
**Other**	10.9%	11.0%	10.9%	10.6%	9.8%	9.7%
**Ethnicity**						
**Non-Hispanic**	96.1%	95.8%**	97.4%	97.4%	97.9%	97.7%
**Hispanic**	3.9%	4.2%	2.6%	2.6%	2.1%	2.3%
**Insurance type**						
**Public payer**	55.9%	57.9%***	76.3%	77.8%**	47.3%	49.2%
**Private payer**	44.1%	42.1%	23.7%	22.2%	52.7%	50.8%
**Age, mean (SD)**	50.0 (19.1)	49.8* (18.8)	47.1 (17.1)	46.7 (17.0)	38.2 (17.2)	37.9 (17.2)
**Age group**						
**10–18**	5.9%	5.1%	2.6%	3.0%	13.8%	14.4%
**19–26**	9.2%	9.1%	12.2%	12.4%	17.4%	17.8%
**27–45**	27.5%	27.5%	33.1%	32.7%	36.1%	35.2%
**46–59**	24.2%	25.3%	26.1%	27.0%	19.7%	19.6%
**60+**	34.1%	33.0%	26.0%	24.9%	13.0%	13.1%

Performed Pearson’s chi-squared test to compare sex, race, ethnicity, insurance type and Welch’s t-test to compare age.

* = Difference comparing PMD with HC had p-value < 0.01.

** = Difference comparing PMD with HC had p-value < 0.001.

*** = Difference comparing PMD with HC had p-value < 0.0001.

### Descriptive patterns of mental healthcare visits

#### Patterns of mental healthcare utilization during and before the COVID-19 pandemic

**Fig 2:**
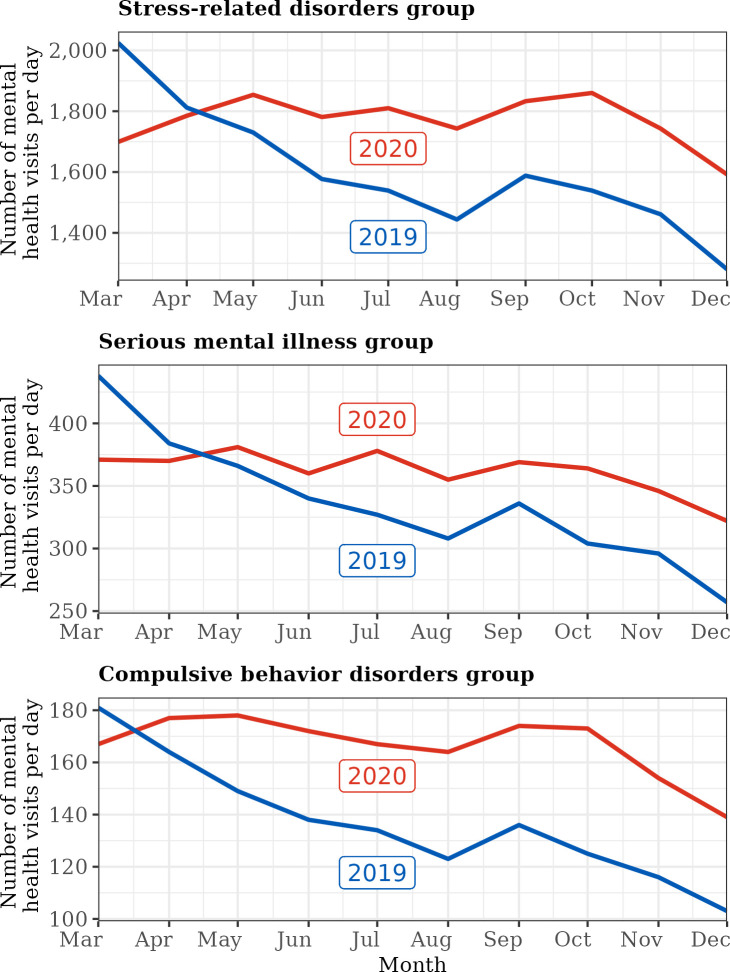
Mean daily number of mental healthcare visits by month among PMDs (red) and HCs (blue). We report mean daily number of mental health visits for each month to account for the variation in duration of months and because our data reporting starts from March 13^th^.

Fig 2 demonstrates the mean daily number of mental healthcare visits by month for the PMD groups in 3/13/2020–12/31/2020 and HCs in 3/13/2019–12/31/2019. The monthly number of mental healthcare visits by PMDs (2020) and HCs (2019) indicated seasonal patterns of overall mental healthcare utilization from year to year. During March and April of 2020 (the peak of the 2020 lockdown), the number of mental healthcare visits were lower compared to the corresponding period in 2019. However, from May onward, the monthly number of mental healthcare visits were greater in 2020 than in 2019, and the increases or decreases in each month were consistent across both years based on visual inspection. Notably, the mean total and quarterly number of mental healthcare visits per patient in 2020 were also significantly greater than that in 2019, especially starting in July (see Table 2).

**Table 2:** Mean total and quarterly number of mental healthcare visits per patient during the pandemic period.

Stress-related disorders group	Mar 13~Dec 31	Mar 13~Jun 30	Jul 1~Sep 31	Oct 1~Dec 31
Mean mental healthcare visits (2020)	4.5	1.7	1.4	1.4
Mean mental healthcare visits (2019)	4.1	1.7	1.2	1.2
p-value	< 0.00001	0.66	< 0.00001	< 0.00001
**Serious mental illness group**				
Mean mental healthcare visits (2020)	9.2	3.5	2.9	2.7
Mean mental healthcare visits (2019)	8.3	3.5	2.5	2.2
p-value	< 0.00001	0.87	< 0.00001	< 0.00001
**Compulsive behavior disorders group**				
Mean mental healthcare visits (2020)	8.3	3.2	2.6	2.4
Mean mental healthcare visits (2019)	7.4	3.2	2.2	2.0
p-value	0.0002	0.53	0.00001	< 0.00001

P values are based on Welch’s t-test, comparing the mean number of mental healthcare visits between 2020 and 2019.

#### Identification of patients with high mental healthcare utilization during the COVID-19 pandemic

**Fig 3:**
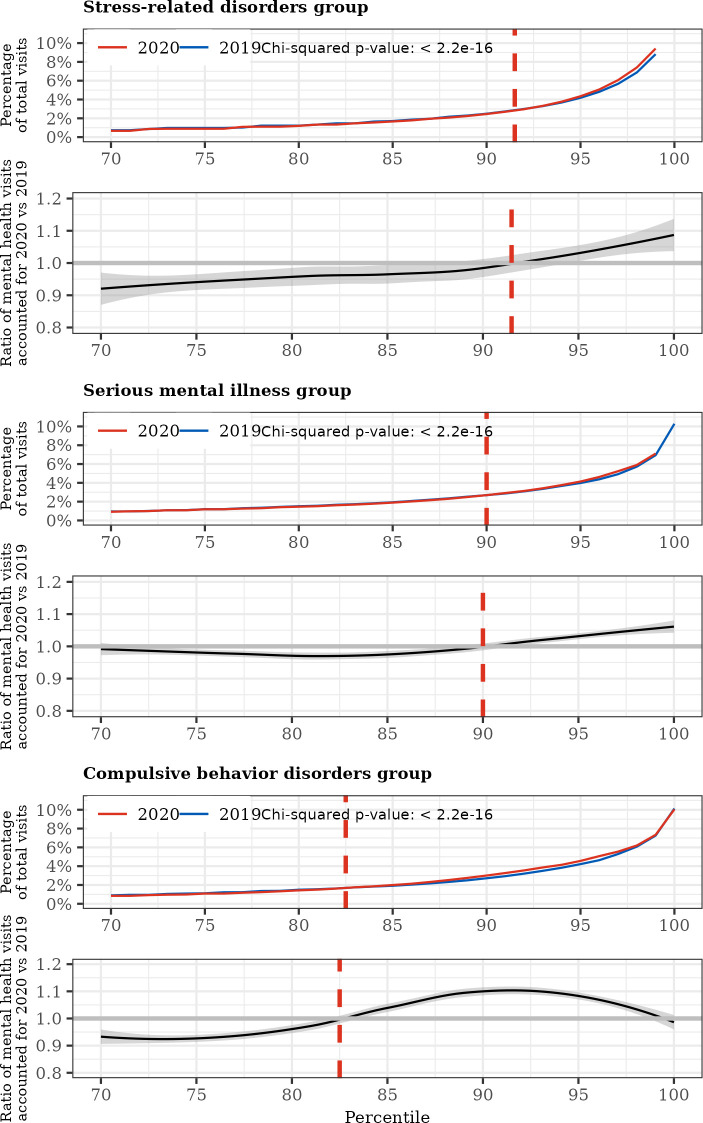
**(Top) proportion of total mental healthcare visits by each mental healthcare visits percentile in PMDs (red) and HCs (blue).** (Bottom) Ratio of the proportion of mental healthcare visits accounted for by each percentile in PMDs to that of the HCs. The top plots represent the distributions of percentages of mental healthcare visits accounted for by each mental healthcare visits percentile of PMDs (red) and HCs (blue). The bottom plots illustrate the percentage of total mental healthcare visits represented by PMDs divided by that of the HCs for each percentile. The dashed red vertical lines indicate the percentiles in which the percentage of total mental healthcare visits represented by PMDs exceed that of HCs.

Fig 3 demonstrates that patients in approximately the top decile of mental healthcare visits accounted for most of the excess visits during the pandemic of 2020 compared to the pre-pandemic period of 2019 (top 8 percentiles for stress-related disorders group, top 10 percentiles for serious mental illness group, and top 17 percentiles for compulsive behavior disorders group).

**Tables 3 and 4** compare the sociodemographic characteristics of the top decile (high utilizers) and bottom nine deciles of mental healthcare utilizers for PMDs (2020) and HCs (2019). For both the PMDs and HCs, a higher proportion of high utilizers were enrolled in public payer insurance compared to the bottom nine deciles across both years. The mean number of mental healthcare visits among the high utilizers were greater during the pandemic period of 2020 than pre-pandemic period of 2019 (stress-related disorders group: 26 for PMD and 22 for HC, *p* < 0.00001; serious mental illness group: 45 for PMD and 39 for HC, *p* < 0.00001; compulsive behavior disorder group: 43 for PMD and 37 for HC, *p* < 0.00001). However, the mean number of non-mental healthcare visits did not differ after Bonferroni correction during vs. before the pandemic (stress-related disorders group: 20.4 for PMD and 19.5 for HC, *p* = 0.001; serious mental illness group: 19.1 for PMD and 17.4 for HC, *p* = 0.02; compulsive behavior disorder group: 17.2 for PMD and 15.8 for HC, *p* = 0.2), suggesting that increases in utilization were relatively isolated to mental healthcare needs.

**Table 3:** Comparison of sociodemographic characteristics of top decile of mental healthcare utilizers vs. the bottom nine deciles among PMDs in 2020.

	Stress-related disorders group (2020)		Serious mental illness group (2020)		Compulsive behavior disorders group (2020)	
	**Top decile**	**Bottom nine deciles**	**Top decile**	**Bottom nine deciles**	**Top decile**	**Bottom nine deciles**
**N**	12,738	103,111	1,265	10,265	642	5,251
**Sex**						
**Female**	66.6%	68.6%***	57.2%	54.5%	68.5%	68.4%
**Male**	33.4%	31.4%	42.8%	45.5%	31.5%	31.6%
**Race**						
**White**	76.9%	82.1%***	80.2%	76.9%	85.0%	81.8%
**Black**	6.0%	4.9%	7.0%	9.7%	3.1%	5.7%
**Asian**	2.1%	2.6%	2.5%	2.5%	2.6%	2.5%
**Other**	15.0%	10.3%	10.3%	11.0%	9.2%	9.9%
**Ethnicity**						
**Non-Hispanic**	94.5%	96.3%***	98.1%	97.3%	98.8%	97.8%
**Hispanic**	5.5%	3.7%	1.9%	2.7%	1.2%	2.2%
**Insurance type**						
**Public payer**	71.3%	54.0%***	80.9%	75.8%***	63.4%	45.3%***
**Private payer**	28.7%	46.0%	19.1%	24.2%	36.6%	54.7%
**Age, mean (SD)**	47.0 (18.9)	50.4 (19.1)***	45.6 (16.6)	47.3 (17.2)**	37.7 (16.7)	38.3 (17.2)
**Age group**						
**10–18**	8.1%	4.7%	3.4%	2.5%	14.2%	13.8%
**19–26**	9.1%	9.2%	12.8%	12.1%	16.8%	17.5%
**27–45**	30.4%	27.1%	33.8%	33.0%	37.7%	35.9%
**46–59**	24.6%	24.1%	27.7%	25.9%	19.8%	19.6%
**60+**	27.8%	34.8%	22.3%	26.4%	11.5%	13.2%
**Mental healthcare visit count, mean**	25.6	1.9***	45.3	4.8***	43.1	4.0***
**Non-mental healthcare visit count, mean**	20.4	12.0***	19.1	11.4***	17.2	10.7***

**Table 4:** Comparison of sociodemographic characteristics of top decile of mental healthcare utilizers vs. the bottom nine deciles among HCs in 2019.

	Stress-related disorders group (2019)		Serious mental illness group (2019)		Compulsive behavior disorders group (2019)	
	**Top decile**	**Bottom nine deciles**	**Top decile**	**Bottom nine deciles**	**Top decile**	**Bottom nine deciles**
**N**	12,496	101,108	1,287	10,471	583	4,804
**Sex**						
**Female**	63.8%	69.5%***	55.9%	54.8%	67.9%	678
**Male**	36.2%	30.5%	44.1%	45.2%	32.1%	322
**Race**						
**White**	77.2%	81.7%***	80.3%	76.9%	87.7%	83.0%*
**Black**	6.0%	5.2%	7.1%	9.9%	1.7%	4.4%
**Asian**	2.5%	2.5%	2.5%	2.5%	3.6%	2.6%
**Other**	14.3%	10.6%	10.2%	10.7%	7.0%	10.0%
**Ethnicity**						
**Non-Hispanic**	94.3%	96.0%***	97.7%	97.3%	98.3%	97.6%
**Hispanic**	5.7%	4.0%	2.3%	2.7%	1.7%	2.4%
**Insurance type**						
**Public payer**	72.9%	56.0%***	82.1%	77.3%***	61.7%	47.7%***
**Private payer**	27.1%	44.0%	17.9%	22.7%	38.3%	52.3%
**Age, mean (SD)**	46.9 (18.8)	50.2 (18.8)***	45.2 (16.6)	46.8 (17.1)**	37.5 (16.9)	38.0 (17.2)
**Age group**						
**10–18**	8.5%	4.7%	4.5%	2.8%	14.9%	14.3%
**19–26**	9.1%	9.1%	13.1%	12.4%	17.3%	17.8%
**27–45**	29.1%	27.3%	33.0%	32.6%	36.9%	35.0%
**46–59**	26.4%	25.2%	28.7%	26.8%	19.0%	19.6%
**60+**	26.9%	33.8%	20.7%	25.4%	11.8%	13.2%
**Mental healthcare visit count, mean**	22.2	1.9***	39.3	4.5***	36.7	3.8***
**Non-mental healthcare visit count, mean**	19.5	11.7***	17.4	10.5***	15.8	10.2***

Performed Pearson’s chi-squared test to compare sex, race, ethnicity, insurance type and Welch’s t-test to compare age, mental healthcare visit count, non-mental healthcare visit count.

* = Difference comparing top decile of mental healthcare utilizers with bottom nine deciles of utilizers p < 0.01

** = Difference comparing top decile of mental healthcare utilizers with bottom nine deciles of utilizers p < 0.001

*** = Difference comparing top decile of mental healthcare utilizers with bottom nine deciles of utilizers p < 0.0001

### Associations of high mental healthcare utilization and clinical history in 2019 and 2020 by disorder groups

#### Stress-related disorders group

**Fig 4:**
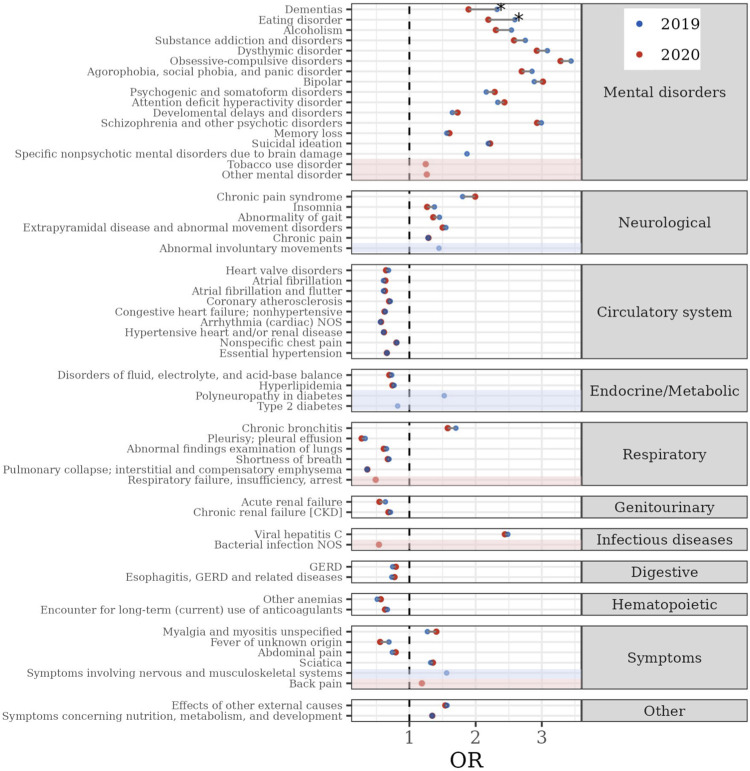
PheWAS comparison plot between stress-related disorders group (PMD) in 2020 and its HC in 2019. The x-axis represents the aOR between each phenotype and high mental healthcare utilization (top decile of utilizer) for the stress-related disorders group (red) and their historical comparison group (HC; blue). The y-axis shows the phenotypes categorized by disease type. The dashed vertical line marks aOR = 1. The red background shows phenotypes that were associated with high mental healthcare utilization only among the stress-related disorders group, and the blue background shows phenotypes that were associated with high mental healthcare utilization only among the HC.

Fig 4 compares the adjusted odds ratio (aOR) of clinical history phenotypes that were significantly associated with high mental healthcare utilization (top decile) in 2019 or 2020 (also see S2 and S3 Tables for more detail). A total of 58 phenotypes (17 mental and 41 non-mental disorder phenotypes) were associated with high mental healthcare utilization in either year, and the majority (48 of 58) had associations in both 2020 and 2019. Of these 48, the aORs for 46 phenotypes were not significantly different between 2020 and 2019, while two phenotypes (dementias (aOR: 2.3 vs. 1.9, *p* = 0.03) and eating disorder (aOR: 2.6 vs. 2.2, *p* = 0.03)) had stronger associations with high mental healthcare utilization in 2019 (see Fig 4, S2 and S3 Tables for details). Tobacco use disorder (aOR = 1.25, 95% CI = 1.18–1.31) was the only mental disorder phenotype associated with high mental healthcare utilization in 2020 only, and back pain (aOR = 1.2, 95% CI = 1.1–1.3) was the only non-mental disorder phenotype associated with high mental healthcare utilization in 2020 only.

Of note, all 17 mental disorder and 6 neurological disorder phenotypes were positively associated while most non-mental disorder phenotypes (26 of 35) were negatively associated with high mental healthcare utilization. The non-mental disorder phenotypes that were positively associated with high mental healthcare utilization in both years included viral hepatitis C (aOR = 2.4, 95% CI = 2.3–2.6 for PMD; aOR = 2.5, 95% CI = 2.4–2.6 for HC), chronic bronchitis (aOR = 1.6, 95% CI = 1.4–1.7 for PMD; aOR = 1.7, 95% CI = 1.6–1.8 for HC), myalgia/myositis (aOR = 1.4, 95% CI = 1.3–1.5 for PMD; aOR = 1.3, 95% CI = 1.2–1.4 for HC), and sciatica (aOR = 1.36, 95% CI = 1.27–1.44 for PMD; aOR = 1.3, 95% CI = 1.2–1.4 for HC).

#### Serious mental illness group

The PheWAS results for the PMD (2020) and HC (2019) with prior serious mental illness are reported in S4 and S5 Tables. While power was relatively limited due to smaller sample sizes, a total of 12 phenotypes (8 mental and 4 non-mental disorder phenotypes) were significantly associated with high mental healthcare utilization in either year. Four of eight mental disorder phenotypes (depression, MDD, generalized anxiety disorder, and PTSD) were associated with high mental healthcare utilization in both 2020 and 2019, and their aORs were not significantly different. Two mental disorder phenotypes (antisocial/borderline personality disorder and OCD) were associated with high mental healthcare utilization in 2020 only (see S2 Fig, S4 and S5 Tables for details). The four non-mental disorder phenotypes were all negatively associated with high mental health care utilization.

#### Compulsive behavior disorders group

The PheWAS results for the PMD (2020) and HC (2019) with prior compulsive behavior disorders are reported in S6 and S7 Tables. Again, while the power was limited, a total of ten phenotypes (2 mental and 8 non-mental disorder phenotypes) were significantly associated with high mental healthcare utilization in either year. Only generalized anxiety disorder was positively associated with high mental healthcare utilization in both 2020 and 2019, and the aOR were not significantly different, and mood disorder was positively associated with high mental healthcare utilization in 2019 (see S3 Fig, S6 and S7 Tables for details). The eight non-mental disorder phenotypes were all negatively associated with high mental health care utilization.

## Discussion

In this study, we leveraged EHR-based data from a large healthcare system to characterize and identify correlates of high mental healthcare utilization during the COVID-19 pandemic in patients with pre-existing mental health conditions. Our descriptive analysis showed that, except for the very beginning stage of the lockdown (March and April of 2020), monthly mental health visits among patients with prior mental disorder were higher during the first nine months of COVID-19 pandemic compared to the previous year.

Our findings are consistent with recent EHR-based studies that reported an increase in mental healthcare utilization in the general population during the pandemic [12, 13]. Given evidence that patients with pre-existing mental disorders experienced exacerbated psychiatric symptoms during the pandemic, it is perhaps unsurprising that their mental healthcare utilization also increased [5, 9, 10, 19]. However, studies on mental healthcare utilization among people with pre-existing mental disorder during the 2002 SARS (severe acute respiratory syndrome) outbreak in Hong Kong and early COVID-19 in Italy suggest that mental healthcare utilization tended to decrease among these individuals during disease pandemics [5, 14]. One reason for this difference may be the expansion of telemedicine services in the United States during the COVID-19 pandemic. Many health systems in the United States experienced a fast recovery to normal levels and then a surge in mental healthcare visit volumes following the COVID-19 pandemic lockdown, mostly driven by the incorporation of telemedicine services [12, 13]. The rapidly increased accessibility of telemedicine services was largely due to federal relaxation of HIPAA compliance and expansion of Medicare coverage for telemedicine [20]. Also, the State of Massachusetts rapidly instituted a temporary policy that prevented termination of individual-level Medicaid coverage during the national emergency that likely benefited patients who sought care at MGB. Thus, our findings may reflect both increased mental health difficulties and increased accessibility of mental health services.

We also found that a subset of high mental healthcare utilizers (approximately the top decile) accounted for most of the excess visits during the first nine months of COVID-19 pandemic compared to the previous year. These high mental healthcare utilizers tended to be on public payer insurance and had substantially greater mental and non-mental healthcare utilization than others. Of note, high mental healthcare utilizers during the first nine months of COVID-19 did not have more non-mental healthcare visits compared to those during the previous year despite the excess mental healthcare visits. This suggests that among patients with pre-existing mental disorder, greater healthcare utilization was likely a function of mental health-specific concerns rather than general health service-seeking.

Our findings also have implications for prioritization of healthcare services by identifying the subgroup of at-risk patients most likely to require excess mental healthcare needs during a pandemic. The PheWAS analysis showed that, despite increased overall utilization of mental healthcare among patients with prior stress-related disorders, factors associated with high mental healthcare utilization were largely similar during and before the pandemic. Unexpectedly, we did not find positive associations between underlying conditions that were reported to pose risk for severe COVID-19 outcomes and high mental healthcare utilization. Additionally, all neurological and mental disorder phenotypes below the significance threshold were associated with high mental healthcare utilization while few non-mental disorder phenotypes (e.g., viral hepatitis C, chronic bronchitis, sciatica, myalgia/myositis, back pain) were. Prior studies have found that viral hepatitis C, chronic bronchitis, and musculoskeletal pain-related conditions (sciatica, myalgia/myositis, back pain) often co-occur with psychiatric disorders [21–25], which may account for these associations. However, back pain was the only non-mental disorder phenotype that was associated with increased risk of high mental healthcare utilization specifically during the pandemic period. Interestingly, several studies have reported increased back pain during the COVID-19 pandemic [26–29]. The heightened occurrence of back pain could plausibly be attributed to decreased physical activity and heightened engagement in sedentary behaviors stemming from isolation measures during the pandemic. Additional research may be needed to explore whether there are causal links between mental health conditions among patients with back pain during the pandemic. For patients with prior serious mental illness or compulsive behavior disorders, a smaller number of clinical phenotypes were associated with high utilization, possibly due to reduced power.

Strengths of this study include the large sample sizes and phenome-wide data that enabled PheWAS analysis leveraging large-scale structured EHR data. In addition, in contrast to most prior studies that have focused on specific types of pre-existing psychiatric conditions, we examined multiple patient cohorts that captured a broad spectrum of psychiatric conditions. However, our study is subject to several limitations. First, because structured EHR data do not include information on several social determinants of health, we were not able to examine several risk factors that have been previously shown to influence mental health during the COVID-19 pandemic (i.e. social support, discrimination) [30–33]. Secondly, our data did not include information on whether visits were virtual or in-person, precluding examination about the impact of telehealth on utilization. Moreover, our data did not include information on whether the visits were self-initiated or scheduled appointments by the providers. Lastly, smaller sample sizes for those with prior serious mental illness and compulsive behavior disorders limited the power of analyses for these groups.

In conclusion, using EHR data from a large US-based healthcare system, we found heightened mental healthcare utilization during the COVID-19 pandemic among patients with recent pre-existing psychiatric disorders, which was largely accounted for by a relatively small subset of patients. We identified several comorbidities (e.g., dementia, eating disorder, tobacco use disorder, back pain) that were differentially associated with high mental healthcare utilization before and after the COVID-19 lockdown, although most clinical correlates were similar during these periods. Overall, these results provide insights into drivers of mental healthcare utilization during the pandemic that may inform efforts to optimize resource prioritization in future pandemic crises.

## Supporting information

S1 Fig Venn diagram of patients who were recently seen in the hospital system for each pre-existing mental disorder (PMD) category. Patients could belong to multiple PMDs as long as they had the relevant ICD-10/phecodes. Vast majority (92%) of patients were exclusive to the stress-related disorders group. However, only 52% and 31% of patients were exclusive to the serious mental illness group and compulsive behavior disorder group, respectively.
(TIF)

S2 Fig PheWAS comparison plot between serious mental illness group and its historical comparison group. The x-axis represents the aOR between each phenotype and high mental healthcare utilization (top decile of utilizer) for the serious mental illness and compulsive behavior disorders groups (red) and their historical comparison groups (HC; blue). The y-axis shows the phenotypes categorized by disease type. The dashed vertical line marks aOR = 1.
(TIF)

S3 Fig PheWAS comparison plot between compulsive behavior disorder group and its historical comparison group. The x-axis represents the aOR between each phenotype and high mental healthcare utilization (top decile of utilizer) for the serious mental illness and compulsive behavior disorders groups (red) and their historical comparison groups (HC; blue). The y-axis shows the phenotypes categorized by disease type. The dashed vertical line marks aOR = 1.
(TIF)

S1 Table List of phecodes pertaining to each pre-existing disorders group. Body dysmorphic disorder is grouped under phecode:303.4 (Somatoform disorder) which includes a variety of other conditions. We identified patients with prior body dysmorphic disorder using ICD-10 code to avoid selecting patients who had other somatoform disorders to our psychiatric conditions group.
(DOCX)

S2 Table PheWAS summary statistics—clinical history phenotypes significantly associated with high mental healthcare utilization among patients with prior stress-related disorder patients (2020). (DOCX)

S3 Table PheWAS summary statistics—clinical history phenotypes significantly associated with high mental healthcare utilization among patients with prior stress-related disorder patients (2019). (DOCX)

S4 Table PheWAS summary statistics—clinical history phenotypes significantly associated with high mental healthcare utilization among patients with prior serious mental illness patients (2020). (DOCX)

S5 Table PheWAS summary statistics—clinical history phenotypes significantly associated with high mental healthcare utilization among patients with prior serious mental illness patients (2019). (DOCX)

S6 Table PheWAS summary statistics—clinical history phenotypes significantly associated with high mental healthcare utilization among patients with prior compulsive behavior disorder patients (2020). (DOCX)

S7 Table PheWAS summary statistics—clinical history phenotypes significantly associated with high mental healthcare utilization among patients with prior compulsive behavior disorder patients (2019). (DOCX)
