# Supplementary figures and images for: Patterns and correlates of mental healthcare utilization during the COVID-19 pandemic among individuals with pre-existing mental disorder
Source: PLoS One. 2024 Jun 4;19(6):e0303079. doi: 10.1371/journal.pone.0303079 (PMC11149861; doi:10.1371/journal.pone.0303079)

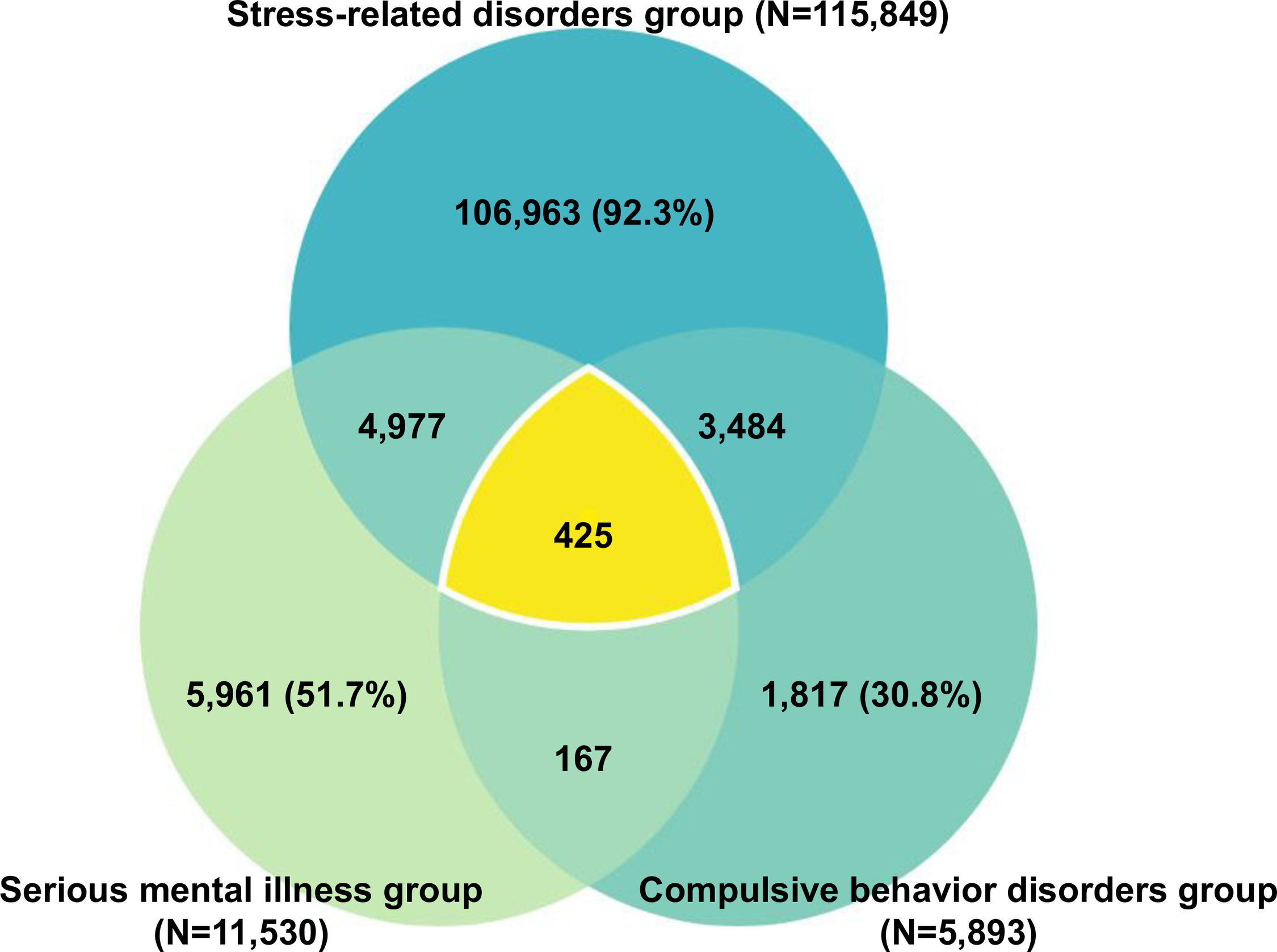

Supplement: S1 Fig — Patients could belong to multiple PMDs as long as they had the relevant ICD-10/phecodes. Vast majority (92%) of patients were exclusive to the stress-related disorders group. However, only 52% and 31% of patients were exclusive to the serious mental illness group and compulsive behavior disorder group, respectively. (TIF) [file pone.0303079.s001.tif]

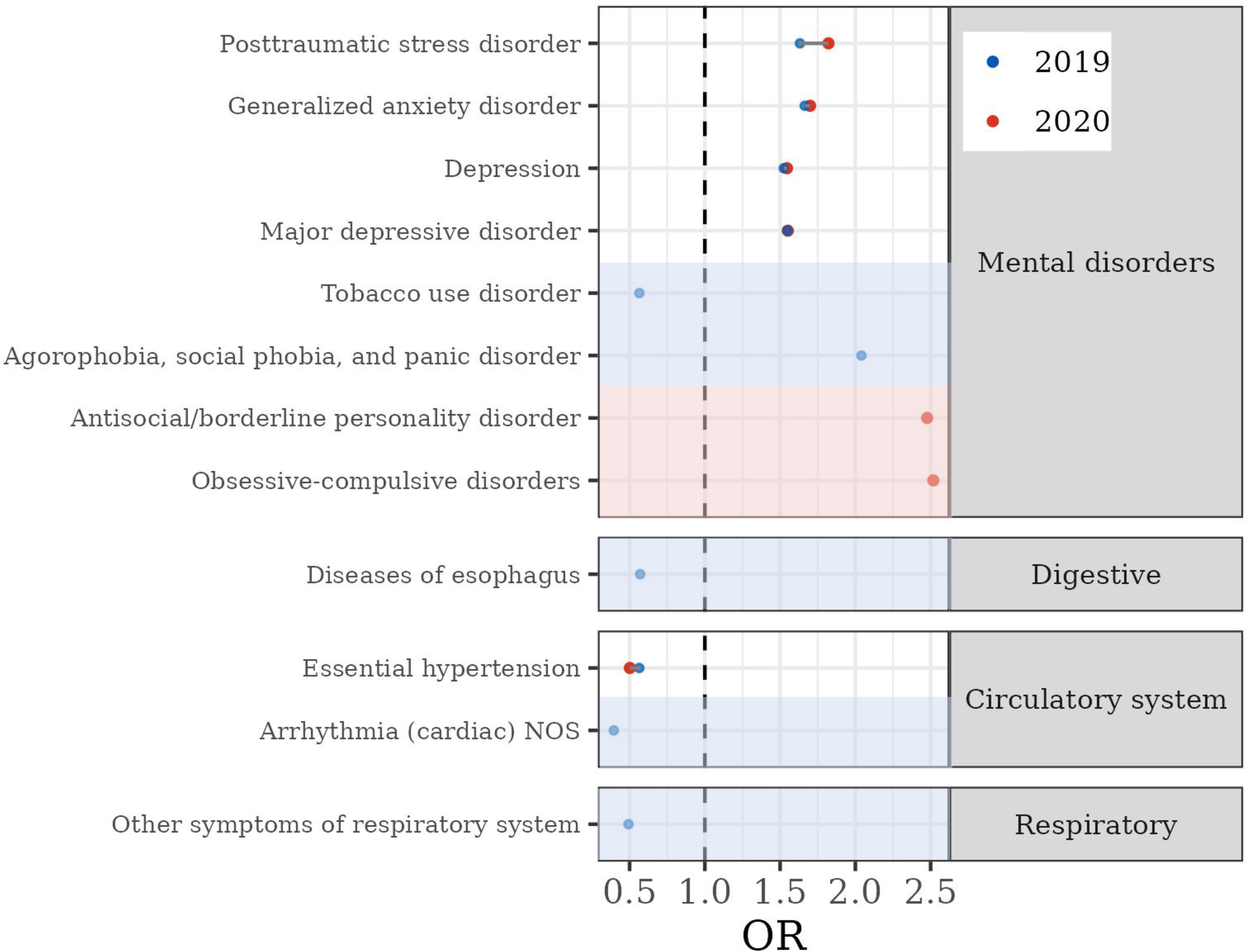

Supplement: S2 Fig — The x-axis represents the aOR between each phenotype and high mental healthcare utilization (top decile of utilizer) for the serious mental illness and compulsive behavior disorders groups (red) and their historical comparison groups (HC; blue). The y-axis shows the phenotypes categorized by disease type. The dashed vertical line marks aOR = 1. (TIF) [file pone.0303079.s002.tif]

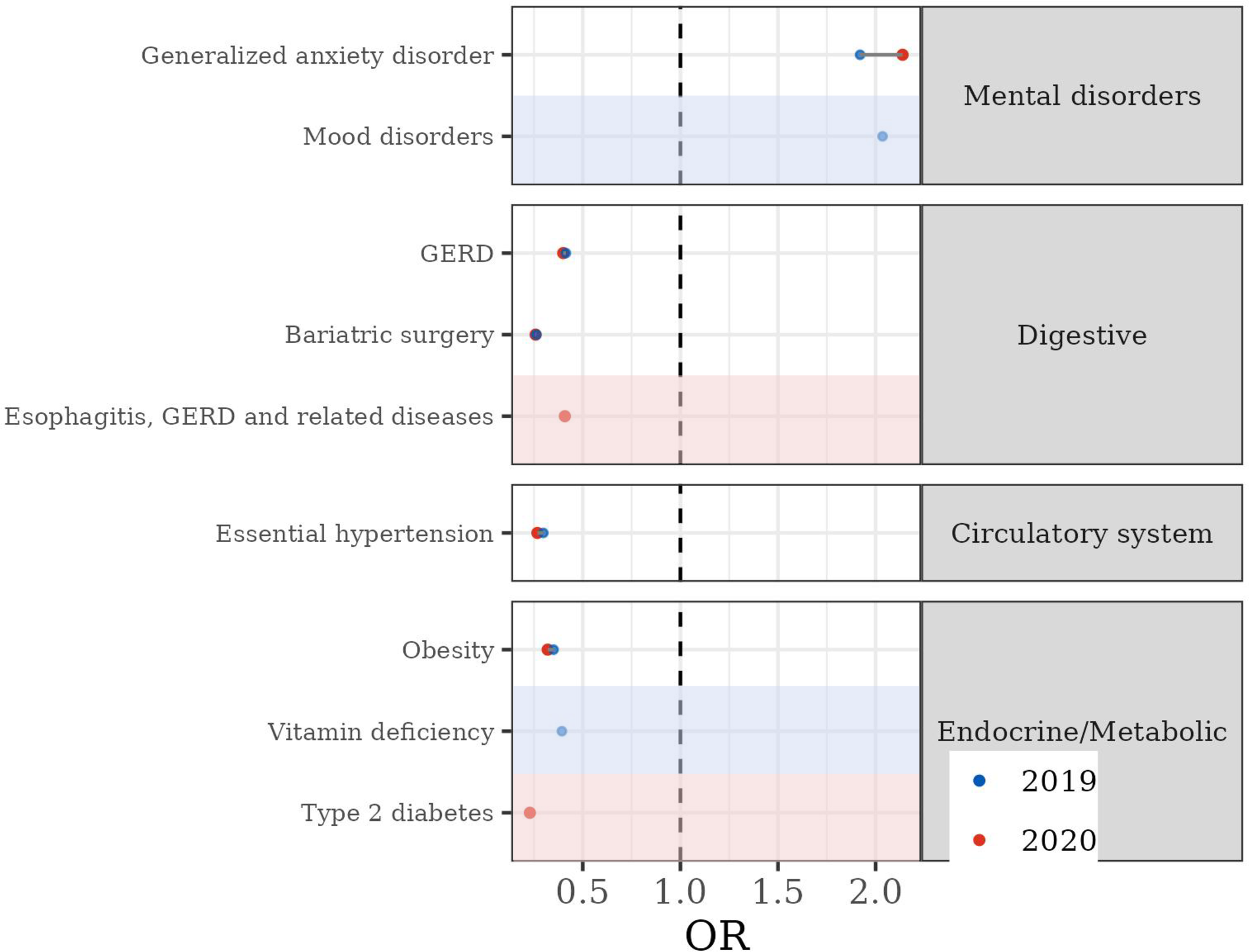

Supplement: S3 Fig — The x-axis represents the aOR between each phenotype and high mental healthcare utilization (top decile of utilizer) for the serious mental illness and compulsive behavior disorders groups (red) and their historical comparison groups (HC; blue). The y-axis shows the phenotypes categorized by disease type. The dashed vertical line marks aOR = 1. (TIF) [file pone.0303079.s003.tif]
