# Supplementary material for: Patterns and correlates of mental healthcare utilization during the COVID-19 pandemic among individuals with pre-existing mental disorder
Source: PLoS One. 2024 Jun 4;19(6):e0303079. doi: 10.1371/journal.pone.0303079 (PMC11149861; doi:10.1371/journal.pone.0303079)
Supplement: S1 Table — Body dysmorphic disorder is grouped under phecode:303.4 (Somatoform disorder) which includes a variety of other conditions. We identified patients with prior body dysmorphic disorder using ICD-10 code to avoid selecting patients who had other somatoform disorders to our psychiatric conditions group. (DOCX) [file pone.0303079.s004.docx]

| **Group** | **Phecodes** |
| --- | --- |
| Stress-related disorders group | - Major Depressive Disorder (phecode: 296.22) - Anxiety Disorders (phecode: 300, 300.1, 300.11) - Acute reaction to stress (phecode: 300.8) - Posttraumatic stress disorder (phecode: 300.9) - Adjustment reaction (phecode: 304) |
| Serious mental illness group | - Schizophrenia (phecode: 295.1) - Psychosis (phecode: 295.3) - Bipolar disorders (phecode: 296.1) |
| Compulsive behavior disorders group | - Obsessive-compulsive disorders (phecode: 300.3) - Eating disorder (phecode: 305) - Body dysmorphic disorder (ICD-10 code: F45.22) |
