# Supplementary material for: Patterns and correlates of mental healthcare utilization during the COVID-19 pandemic among individuals with pre-existing mental disorder
Source: PLoS One. 2024 Jun 4;19(6):e0303079. doi: 10.1371/journal.pone.0303079 (PMC11149861; doi:10.1371/journal.pone.0303079)
Supplement: S2 Table — (DOCX) [file pone.0303079.s005.docx]

| **Phenotype** | **Description** | **Category** | **OR** | **SE** | **p.bonferroni** | **n_total** | **n_cases** | **n_controls** |
| --- | --- | --- | --- | --- | --- | --- | --- | --- |
| 041 | Bacterial infection NOS | infectious diseases | 0.539 | 0.085 | 1.01E-10 | 112105 | 1548 | 110557 |
| 070 | Viral hepatitis | infectious diseases | 2.170 | 0.061 | 8.07E-34 | 113724 | 1464 | 112260 |
| 070.3 | Viral hepatitis C | infectious diseases | 2.441 | 0.065 | 3.29E-40 | 113789 | 1231 | 112558 |
| 250 | Diabetes mellitus | endocrine/metabolic | 0.820 | 0.034 | 2.59E-06 | 112965 | 11282 | 101683 |
| 272 | Disorders of lipoid metabolism | endocrine/metabolic | 0.742 | 0.028 | 1.17E-24 | 108570 | 30272 | 78298 |
| 272.1 | Hyperlipidemia | endocrine/metabolic | 0.742 | 0.028 | 1.30E-24 | 108572 | 30247 | 78325 |
| 276 | Disorders of fluid, electrolyte, and acid-base balance | endocrine/metabolic | 0.695 | 0.045 | 3.95E-13 | 111669 | 5888 | 105781 |
| 285 | Other anemias | hematopoietic | 0.566 | 0.046 | 2.35E-32 | 111293 | 6411 | 104882 |
| 286.2 | Encounter for long-term (current) use of anticoagulants | hematopoietic | 0.630 | 0.066 | 1.37E-09 | 112845 | 2980 | 109865 |
| 290 | Delirium dementia and amnestic and other cognitive disorders | mental disorders | 1.741 | 0.064 | 2.08E-15 | 113204 | 2064 | 111140 |
| 290.1 | Dementias | mental disorders | 1.893 | 0.082 | 2.88E-12 | 113783 | 1368 | 112415 |
| 291 | Other specified nonpsychotic and/or transient mental disorders | mental disorders | 1.756 | 0.063 | 2.66E-16 | 113420 | 1635 | 111785 |
| 292 | Neurological disorders | mental disorders | 1.401 | 0.035 | 4.66E-19 | 112083 | 7420 | 104663 |
| 292.3 | Memory loss | mental disorders | 1.603 | 0.058 | 2.35E-13 | 113561 | 2529 | 111032 |
| 295 | Schizophrenia and other psychotic disorders | mental disorders | 2.929 | 0.054 | 4.49E-86 | 113739 | 1767 | 111972 |
| 296 | Mood disorders | mental disorders | 2.218 | 0.023 | 3.84E-261 | 103249 | 51797 | 51452 |
| 296.1 | Bipolar | mental disorders | 3.017 | 0.034 | 6.11E-232 | 112964 | 5117 | 107847 |
| 297 | Suicidal ideation or attempt | mental disorders | 2.168 | 0.047 | 1.90E-58 | 113125 | 2666 | 110459 |
| 297.1 | Suicidal ideation | mental disorders | 2.218 | 0.048 | 3.77E-60 | 113142 | 2553 | 110589 |
| 300.12 | Agoraphobia, social phobia, and panic disorder | mental disorders | 2.695 | 0.037 | 1.86E-152 | 113042 | 4253 | 108789 |
| 300.3 | Obsessive-compulsive disorders | mental disorders | 3.283 | 0.065 | 9.14E-72 | 113749 | 1231 | 112518 |
| 300.4 | Dysthymic disorder | mental disorders | 2.925 | 0.061 | 2.18E-67 | 113870 | 1505 | 112365 |
| 303 | Psychogenic and somatoform disorders | mental disorders | 2.287 | 0.068 | 5.10E-31 | 113842 | 1234 | 112608 |
| 305.2 | Eating disorder | mental disorders | 2.192 | 0.056 | 2.37E-42 | 113706 | 2017 | 111689 |
| 306 | Other mental disorder | mental disorders | 1.263 | 0.041 | 4.45E-06 | 111651 | 5062 | 106589 |
| 313 | Pervasive developmental disorders | mental disorders | 2.414 | 0.030 | 2.65E-186 | 112425 | 9026 | 103399 |
| 313.1 | Attention deficit hyperactivity disorder | mental disorders | 2.434 | 0.033 | 2.77E-159 | 112697 | 6874 | 105823 |
| 315 | Developmental delays and disorders | mental disorders | 1.727 | 0.057 | 3.05E-19 | 113437 | 1871 | 111566 |
| 316 | Substance addiction and disorders | mental disorders | 2.579 | 0.032 | 1.19E-188 | 111733 | 6212 | 105521 |
| 317 | Alcohol-related disorders | mental disorders | 2.483 | 0.035 | 3.61E-148 | 112724 | 5436 | 107288 |
| 317.1 | Alcoholism | mental disorders | 2.305 | 0.043 | 3.03E-82 | 112902 | 3364 | 109538 |
| 318 | Tobacco use disorder | mental disorders | 1.246 | 0.027 | 5.55E-14 | 106650 | 16462 | 90188 |
| 327 | Sleep disorders | neurological | 1.295 | 0.029 | 7.44E-17 | 110206 | 12886 | 97320 |
| 327.4 | Insomnia | neurological | 1.268 | 0.034 | 5.96E-10 | 111115 | 9001 | 102114 |
| 333 | Extrapyramidal disease and abnormal movement disorders | neurological | 1.502 | 0.071 | 4.30E-06 | 113818 | 1485 | 112333 |
| 338.2 | Chronic pain | neurological | 1.287 | 0.031 | 5.41E-14 | 106917 | 10781 | 96136 |
| 350.2 | Abnormality of gait | neurological | 1.359 | 0.054 | 5.22E-06 | 113120 | 3166 | 109954 |
| 355.1 | Chronic pain syndrome | neurological | 1.993 | 0.058 | 3.29E-30 | 113840 | 1821 | 112019 |
| 375.1 | Dry eyes | sense organs | 1.460 | 0.068 | 9.70E-06 | 113779 | 1695 | 112084 |
| 395 | Heart valve disorders | circulatory system | 0.645 | 0.066 | 1.21E-08 | 112309 | 3207 | 109102 |
| 401 | Hypertension | circulatory system | 0.618 | 0.028 | 5.50E-62 | 110161 | 34293 | 75868 |
| 401.1 | Essential hypertension | circulatory system | 0.658 | 0.028 | 7.11E-48 | 110306 | 33697 | 76609 |
| 401.2 | Hypertensive heart and/or renal disease | circulatory system | 0.615 | 0.066 | 6.34E-11 | 111895 | 3193 | 108702 |
| 411 | Ischemic Heart Disease | circulatory system | 0.660 | 0.044 | 9.16E-19 | 110457 | 8073 | 102384 |
| 411.4 | Coronary atherosclerosis | circulatory system | 0.692 | 0.049 | 2.68E-11 | 112986 | 6420 | 106566 |
| 418 | Nonspecific chest pain | circulatory system | 0.802 | 0.034 | 2.05E-08 | 109653 | 10740 | 98913 |
| 427 | Cardiac dysrhythmias | circulatory system | 0.585 | 0.031 | 2.18E-63 | 107957 | 17642 | 90315 |
| 427.2 | Atrial fibrillation and flutter | circulatory system | 0.629 | 0.058 | 3.75E-13 | 113514 | 4799 | 108715 |
| 427.21 | Atrial fibrillation | circulatory system | 0.636 | 0.059 | 5.03E-12 | 113488 | 4583 | 108905 |
| 427.5 | Arrhythmia (cardiac) NOS | circulatory system | 0.570 | 0.052 | 5.90E-25 | 110452 | 4950 | 105502 |
| 428 | Congestive heart failure; nonhypertensive | circulatory system | 0.623 | 0.064 | 7.50E-11 | 113472 | 3253 | 110219 |
| 496.2 | Chronic bronchitis | respiratory | 1.579 | 0.069 | 1.97E-08 | 113813 | 1565 | 112248 |
| 507 | Pleurisy; pleural effusion | respiratory | 0.277 | 0.099 | 1.83E-35 | 113231 | 1905 | 111326 |
| 508 | Pulmonary collapse; interstitial and compensatory emphysema | respiratory | 0.363 | 0.091 | 1.71E-26 | 112625 | 1947 | 110678 |
| 509 | Respiratory failure, insufficiency, arrest | respiratory | 0.489 | 0.096 | 2.90E-11 | 113430 | 1321 | 112109 |
| 512 | Other symptoms of respiratory system | respiratory | 0.718 | 0.029 | 3.43E-28 | 108216 | 19199 | 89017 |
| 512.7 | Shortness of breath | respiratory | 0.672 | 0.041 | 1.07E-19 | 110788 | 7471 | 103317 |
| 514 | Abnormal findings examination of lungs | respiratory | 0.612 | 0.047 | 4.49E-23 | 110775 | 6349 | 104426 |
| 530 | Diseases of esophagus | digestive | 0.759 | 0.028 | 7.57E-20 | 108161 | 19180 | 88981 |
| 530.1 | Esophagitis, GERD and related diseases | digestive | 0.774 | 0.028 | 7.48E-17 | 108207 | 18601 | 89606 |
| 530.11 | GERD | digestive | 0.794 | 0.029 | 1.39E-12 | 108189 | 16690 | 91499 |
| 585 | Renal failure | genitourinary | 0.612 | 0.047 | 3.12E-23 | 112752 | 6760 | 105992 |
| 585.1 | Acute renal failure | genitourinary | 0.546 | 0.071 | 5.99E-15 | 113200 | 2494 | 110706 |
| 585.3 | Chronic renal failure [CKD] | genitourinary | 0.681 | 0.053 | 1.35E-10 | 113229 | 5019 | 108210 |
| 703 | Diseases of nail, NOS | dermatologic | 1.714 | 0.075 | 3.39E-10 | 113910 | 1233 | 112677 |
| 706.8 | Other specified diseases of sebaceous glands | dermatologic | 1.519 | 0.073 | 4.90E-06 | 113907 | 1427 | 112480 |
| 760 | Back pain | symptoms | 1.188 | 0.029 | 1.02E-06 | 110026 | 12620 | 97406 |
| 764 | Sciatica | symptoms | 1.355 | 0.043 | 8.26E-10 | 113207 | 4576 | 108631 |
| 770 | Myalgia and myositis unspecified | symptoms | 1.406 | 0.056 | 6.10E-07 | 113602 | 2357 | 111245 |
| 783 | Fever of unknown origin | symptoms | 0.557 | 0.063 | 8.44E-18 | 112495 | 2916 | 109579 |
| 785 | Abdominal pain | symptoms | 0.792 | 0.029 | 1.25E-13 | 109597 | 15783 | 93814 |
| 1002 | Symptoms concerning nutrition, metabolism, and development | other | 1.345 | 0.050 | 1.03E-06 | 113262 | 3215 | 110047 |
| 1015 | Effects of other external causes | other | 1.542 | 0.060 | 1.57E-10 | 112096 | 2016 | 110080 |
