# Supplementary material for: Patterns and correlates of mental healthcare utilization during the COVID-19 pandemic among individuals with pre-existing mental disorder
Source: PLoS One. 2024 Jun 4;19(6):e0303079. doi: 10.1371/journal.pone.0303079 (PMC11149861; doi:10.1371/journal.pone.0303079)
Supplement: S3 Table — (DOCX) [file pone.0303079.s006.docx]

| **Phenotype** | **Description** | **Category** | **OR** | **SE** | **p.bonferroni** | **n_total** | **n_cases** | **n_controls** |
| --- | --- | --- | --- | --- | --- | --- | --- | --- |
| 070 | Viral hepatitis | infectious diseases | 2.233 | 0.057 | 5.52E-43 | 111078 | 1702 | 109376 |
| 070.3 | Viral hepatitis C | infectious diseases | 2.484 | 0.060 | 3.90E-49 | 111173 | 1441 | 109732 |
| 197 | Chemotherapy | neoplasms | 0.726 | 0.056 | 3.37E-06 | 109982 | 3736 | 106246 |
| 250 | Diabetes mellitus | endocrine/metabolic | 0.813 | 0.034 | 6.73E-07 | 110386 | 10915 | 99471 |
| 250.2 | Type 2 diabetes | endocrine/metabolic | 0.821 | 0.035 | 8.09E-06 | 110367 | 10431 | 99936 |
| 250.6 | Polyneuropathy in diabetes | endocrine/metabolic | 1.526 | 0.075 | 7.74E-06 | 111228 | 1259 | 109969 |
| 272 | Disorders of lipoid metabolism | endocrine/metabolic | 0.768 | 0.028 | 1.27E-18 | 105759 | 29258 | 76501 |
| 272.1 | Hyperlipidemia | endocrine/metabolic | 0.766 | 0.028 | 6.29E-19 | 105773 | 29238 | 76535 |
| 276 | Disorders of fluid, electrolyte, and acid-base balance | endocrine/metabolic | 0.730 | 0.046 | 1.88E-09 | 109405 | 5516 | 103889 |
| 285 | Other anemias | hematopoietic | 0.512 | 0.047 | 5.23E-43 | 108540 | 6247 | 102293 |
| 286.2 | Encounter for long-term (current) use of anticoagulants | hematopoietic | 0.666 | 0.067 | 4.26E-07 | 110358 | 2695 | 107663 |
| 290 | Delirium dementia and amnestic and other cognitive disorders | mental disorders | 1.972 | 0.063 | 2.26E-24 | 110745 | 1973 | 108772 |
| 290.1 | Dementias | mental disorders | 2.322 | 0.078 | 1.33E-24 | 111203 | 1331 | 109872 |
| 291 | Other specified nonpsychotic and/or transient mental disorders | mental disorders | 1.683 | 0.062 | 1.78E-14 | 110886 | 1731 | 109155 |
| 291.4 | Specific nonpsychotic mental disorders due to brain damage | mental disorders | 1.869 | 0.074 | 1.13E-14 | 111291 | 1174 | 110117 |
| 292 | Neurological disorders | mental disorders | 1.359 | 0.037 | 2.05E-14 | 109644 | 6724 | 102920 |
| 292.3 | Memory loss | mental disorders | 1.565 | 0.062 | 1.75E-10 | 111052 | 2241 | 108811 |
| 295 | Schizophrenia and other psychotic disorders | mental disorders | 2.992 | 0.053 | 5.58E-94 | 111213 | 1844 | 109369 |
| 296 | Mood disorders | mental disorders | 2.407 | 0.024 | 1.38E-299 | 100531 | 49539 | 50992 |
| 296.1 | Bipolar | mental disorders | 2.886 | 0.033 | 1.81E-225 | 110213 | 5604 | 104609 |
| 297 | Suicidal ideation or attempt | mental disorders | 2.219 | 0.048 | 3.34E-60 | 110606 | 2549 | 108057 |
| 297.1 | Suicidal ideation | mental disorders | 2.190 | 0.049 | 2.49E-55 | 110622 | 2423 | 108199 |
| 300.12 | Agoraphobia, social phobia, and panic disorder | mental disorders | 2.851 | 0.038 | 4.17E-168 | 110512 | 4163 | 106349 |
| 300.3 | Obsessive-compulsive disorders | mental disorders | 3.442 | 0.065 | 6.08E-79 | 111166 | 1245 | 109921 |
| 300.4 | Dysthymic disorder | mental disorders | 3.084 | 0.061 | 1.29E-73 | 111302 | 1475 | 109827 |
| 303 | Psychogenic and somatoform disorders | mental disorders | 2.160 | 0.068 | 8.95E-27 | 111259 | 1278 | 109981 |
| 305.2 | Eating disorder | mental disorders | 2.591 | 0.060 | 1.11E-53 | 111226 | 1609 | 109617 |
| 313 | Pervasive developmental disorders | mental disorders | 2.259 | 0.031 | 1.08E-148 | 109852 | 8531 | 101321 |
| 313.1 | Attention deficit hyperactivity disorder | mental disorders | 2.334 | 0.034 | 1.52E-135 | 110143 | 6487 | 103656 |
| 315 | Developmental delays and disorders | mental disorders | 1.649 | 0.059 | 6.01E-15 | 110950 | 1785 | 109165 |
| 316 | Substance addiction and disorders | mental disorders | 2.749 | 0.032 | 8.16E-214 | 109243 | 6100 | 103143 |
| 317 | Alcohol-related disorders | mental disorders | 2.599 | 0.035 | 2.73E-165 | 110219 | 5291 | 104928 |
| 317.1 | Alcoholism | mental disorders | 2.540 | 0.041 | 7.27E-111 | 110452 | 3462 | 106990 |
| 327 | Sleep disorders | neurological | 1.394 | 0.029 | 6.21E-28 | 107676 | 12115 | 95561 |
| 327.4 | Insomnia | neurological | 1.377 | 0.034 | 1.65E-18 | 108462 | 8382 | 100080 |
| 333 | Extrapyramidal disease and abnormal movement disorders | neurological | 1.550 | 0.074 | 1.12E-06 | 111228 | 1326 | 109902 |
| 338.2 | Chronic pain | neurological | 1.282 | 0.031 | 5.57E-13 | 104536 | 10332 | 94204 |
| 350 | Abnormal movement | neurological | 1.354 | 0.043 | 1.07E-09 | 109877 | 4805 | 105072 |
| 350.1 | Abnormal involuntary movements | neurological | 1.446 | 0.062 | 8.38E-07 | 110745 | 2054 | 108691 |
| 350.2 | Abnormality of gait | neurological | 1.453 | 0.056 | 7.55E-09 | 110621 | 2746 | 107875 |
| 355.1 | Chronic pain syndrome | neurological | 1.803 | 0.059 | 1.02E-20 | 111281 | 1789 | 109492 |
| 395 | Heart valve disorders | circulatory system | 0.684 | 0.065 | 2.34E-06 | 109849 | 2979 | 106870 |
| 401 | Hypertension | circulatory system | 0.616 | 0.029 | 8.80E-62 | 107492 | 33419 | 74073 |
| 401.1 | Essential hypertension | circulatory system | 0.661 | 0.028 | 4.72E-46 | 107598 | 32836 | 74762 |
| 401.2 | Hypertensive heart and/or renal disease | circulatory system | 0.607 | 0.069 | 1.28E-10 | 109631 | 2704 | 106927 |
| 411 | Ischemic Heart Disease | circulatory system | 0.644 | 0.045 | 6.50E-20 | 107722 | 7266 | 100456 |
| 411.4 | Coronary atherosclerosis | circulatory system | 0.709 | 0.050 | 2.96E-09 | 110528 | 5715 | 104813 |
| 418 | Nonspecific chest pain | circulatory system | 0.807 | 0.034 | 1.84E-07 | 107526 | 10029 | 97497 |
| 427 | Cardiac dysrhythmias | circulatory system | 0.562 | 0.032 | 1.38E-68 | 105561 | 16185 | 89376 |
| 427.2 | Atrial fibrillation and flutter | circulatory system | 0.607 | 0.060 | 3.53E-14 | 111060 | 4283 | 106777 |
| 427.21 | Atrial fibrillation | circulatory system | 0.607 | 0.061 | 1.50E-13 | 111026 | 4083 | 106943 |
| 427.5 | Arrhythmia (cardiac) NOS | circulatory system | 0.561 | 0.051 | 1.31E-26 | 108073 | 4911 | 103162 |
| 428 | Congestive heart failure; nonhypertensive | circulatory system | 0.634 | 0.067 | 4.92E-09 | 110930 | 2774 | 108156 |
| 496.2 | Chronic bronchitis | respiratory | 1.701 | 0.071 | 3.44E-11 | 111263 | 1404 | 109859 |
| 507 | Pleurisy; pleural effusion | respiratory | 0.330 | 0.098 | 5.55E-27 | 110706 | 1638 | 109068 |
| 508 | Pulmonary collapse; interstitial and compensatory emphysema | respiratory | 0.360 | 0.092 | 4.63E-26 | 110126 | 1751 | 108375 |
| 512 | Other symptoms of respiratory system | respiratory | 0.709 | 0.030 | 2.03E-28 | 106070 | 17311 | 88759 |
| 512.7 | Shortness of breath | respiratory | 0.690 | 0.044 | 1.50E-14 | 108489 | 6113 | 102376 |
| 514 | Abnormal findings examination of lungs | respiratory | 0.652 | 0.048 | 1.48E-16 | 108626 | 5690 | 102936 |
| 530 | Diseases of esophagus | digestive | 0.716 | 0.029 | 3.83E-28 | 105421 | 18519 | 86902 |
| 530.1 | Esophagitis, GERD and related diseases | digestive | 0.733 | 0.029 | 5.26E-24 | 105466 | 17992 | 87474 |
| 530.11 | GERD | digestive | 0.746 | 0.030 | 5.38E-20 | 105496 | 16344 | 89152 |
| 585 | Renal failure | genitourinary | 0.627 | 0.049 | 2.47E-19 | 110240 | 5776 | 104464 |
| 585.1 | Acute renal failure | genitourinary | 0.635 | 0.070 | 3.28E-08 | 110750 | 2275 | 108475 |
| 585.3 | Chronic renal failure [CKD] | genitourinary | 0.712 | 0.056 | 3.52E-07 | 110662 | 4041 | 106621 |
| 638 | Other high-risk pregnancy | pregnancy complications | 0.369 | 0.118 | 1.03E-14 | 111306 | 1240 | 110066 |
| 690 | Erythematosquamous dermatosis | dermatologic | 1.574 | 0.074 | 2.75E-07 | 111404 | 1336 | 110068 |
| 690.1 | Seborrheic dermatitis | dermatologic | 1.574 | 0.074 | 2.75E-07 | 111404 | 1336 | 110068 |
| 706 | Diseases of sebaceous glands | symptoms | 1.249 | 0.041 | 1.91E-05 | 110526 | 5461 | 105065 |
| 764 | Sciatica | symptoms | 1.321 | 0.044 | 6.00E-08 | 110666 | 4537 | 106129 |
| 770 | Myalgia and myositis unspecified | symptoms | 1.272 | 0.043 | 8.30E-06 | 110465 | 4696 | 105769 |
| 781 | Symptoms involving nervous and musculoskeletal systems | symptoms | 1.562 | 0.077 | 2.35E-06 | 110906 | 1234 | 109672 |
| 783 | Fever of unknown origin | symptoms | 0.689 | 0.061 | 3.52E-07 | 110191 | 2765 | 107426 |
| 785 | Abdominal pain | symptoms | 0.741 | 0.030 | 2.72E-21 | 107564 | 15191 | 92373 |
| 1002 | Symptoms concerning nutrition, metabolism, and development | other | 1.343 | 0.051 | 3.62E-06 | 110863 | 2979 | 107884 |
| 1015 | Effects of other external causes | other | 1.568 | 0.062 | 1.26E-10 | 109554 | 1859 | 107695 |
