# Supplementary material for: Patterns and correlates of mental healthcare utilization during the COVID-19 pandemic among individuals with pre-existing mental disorder
Source: PLoS One. 2024 Jun 4;19(6):e0303079. doi: 10.1371/journal.pone.0303079 (PMC11149861; doi:10.1371/journal.pone.0303079)
Supplement: S4 Table — (DOCX) [file pone.0303079.s007.docx]

| **Phenotype** | **Description** | **Category** | **OR** | **SE** | **p.bonferroni** | **n_total** | **n_cases** | **n_controls** |
| --- | --- | --- | --- | --- | --- | --- | --- | --- |
| 296.2 | Depression | mental disorders | 1.546 | 0.072 | 1.28E-06 | 10475 | 2527 | 7948 |
| 296.22 | Major depressive disorder | mental disorders | 1.551 | 0.072 | 1.09E-06 | 10474 | 2484 | 7990 |
| 300.11 | Generalized anxiety disorder | mental disorders | 1.699 | 0.084 | 2.45E-07 | 11042 | 1250 | 9792 |
| 300.3 | Obsessive-compulsive disorders | mental disorders | 2.518 | 0.152 | 1.36E-06 | 11208 | 250 | 10958 |
| 300.9 | Posttraumatic stress disorder | mental disorders | 1.821 | 0.081 | 1.27E-10 | 10941 | 1413 | 9528 |
| 301 | Personality disorders | mental disorders | 2.125 | 0.129 | 5.48E-06 | 11132 | 372 | 10760 |
| 301.2 | Antisocial/borderline personality disorder | mental disorders | 2.477 | 0.146 | 5.52E-07 | 11196 | 272 | 10924 |
| 401 | Hypertension | circulatory system | 0.455 | 0.093 | 3.02E-14 | 10750 | 2855 | 7895 |
| 401.1 | Essential hypertension | circulatory system | 0.502 | 0.092 | 7.00E-11 | 10776 | 2780 | 7996 |
