# Supplementary material for: Patterns and correlates of mental healthcare utilization during the COVID-19 pandemic among individuals with pre-existing mental disorder
Source: PLoS One. 2024 Jun 4;19(6):e0303079. doi: 10.1371/journal.pone.0303079 (PMC11149861; doi:10.1371/journal.pone.0303079)
Supplement: S5 Table — (DOCX) [file pone.0303079.s008.docx]

| **Phenotype** | **Description** | **Category** | **OR** | **SE** | **p.bonferroni** | **n_total** | **n_cases** | **n_controls** |
| --- | --- | --- | --- | --- | --- | --- | --- | --- |
| 296.2 | Depression | mental disorders | 1.525 | 0.072 | 4.81E-06 | 10681 | 2556 | 8125 |
| 296.22 | Major depressive disorder | mental disorders | 1.549 | 0.072 | 1.31E-06 | 10677 | 2523 | 8154 |
| 300.11 | Generalized anxiety disorder | mental disorders | 1.664 | 0.087 | 4.92E-06 | 11235 | 1185 | 10050 |
| 300.12 | Agoraphobia, social phobia, and panic disorder | mental disorders | 2.040 | 0.118 | 1.41E-06 | 11353 | 497 | 10856 |
| 300.9 | Posttraumatic stress disorder | mental disorders | 1.632 | 0.084 | 5.78E-06 | 11156 | 1368 | 9788 |
| 318 | Tobacco use disorder | mental disorders | 0.565 | 0.080 | 1.05E-09 | 10406 | 3030 | 7376 |
| 401 | Hypertension | circulatory system | 0.539 | 0.090 | 8.37E-09 | 10992 | 2941 | 8051 |
| 401.1 | Essential hypertension | circulatory system | 0.564 | 0.090 | 2.11E-07 | 11006 | 2849 | 8157 |
| 427 | Cardiac dysrhythmias | circulatory system | 0.533 | 0.099 | 2.46E-07 | 10412 | 1679 | 8733 |
| 427.5 | Arrhythmia (cardiac) NOS | circulatory system | 0.395 | 0.159 | 5.80E-06 | 10842 | 564 | 10278 |
| 512 | Other symptoms of respiratory system | respiratory | 0.493 | 0.102 | 3.92E-09 | 10853 | 1614 | 9239 |
| 530 | Diseases of esophagus | digestive | 0.570 | 0.098 | 1.06E-05 | 10952 | 1633 | 9319 |
