# Supplementary material for: Patterns and correlates of mental healthcare utilization during the COVID-19 pandemic among individuals with pre-existing mental disorder
Source: PLoS One. 2024 Jun 4;19(6):e0303079. doi: 10.1371/journal.pone.0303079 (PMC11149861; doi:10.1371/journal.pone.0303079)
Supplement: S6 Table — (DOCX) [file pone.0303079.s009.docx]

| **Phenotype** | **Description** | **Category** | **OR** | **SE** | **p.bonferroni** | **n_total** | **n_cases** | **n_controls** |
| --- | --- | --- | --- | --- | --- | --- | --- | --- |
| 250 | Diabetes mellitus | endocrine/metabolic | 0.236 | 0.234 | 5.25E-07 | 5699 | 415 | 5284 |
| 250.2 | Type 2 diabetes | endocrine/metabolic | 0.229 | 0.242 | 9.32E-07 | 5702 | 393 | 5309 |
| 278 | Overweight, obesity and other hyperalimentation | endocrine/metabolic | 0.338 | 0.122 | 5.63E-16 | 5566 | 1900 | 3666 |
| 278.1 | Obesity | endocrine/metabolic | 0.320 | 0.128 | 4.79E-16 | 5616 | 1711 | 3905 |
| 278.11 | Morbid obesity | endocrine/metabolic | 0.261 | 0.149 | 1.82E-16 | 5641 | 1350 | 4291 |
| 300.11 | Generalized anxiety disorder | mental disorders | 2.138 | 0.099 | 1.68E-11 | 5593 | 1298 | 4295 |
| 401 | Hypertension | circulatory system | 0.235 | 0.171 | 2.24E-14 | 5605 | 977 | 4628 |
| 401.1 | Essential hypertension | circulatory system | 0.267 | 0.168 | 4.04E-12 | 5610 | 959 | 4651 |
| 530 | Diseases of esophagus | digestive | 0.393 | 0.141 | 3.08E-08 | 5487 | 1055 | 4432 |
| 530.1 | Esophagitis, GERD and related diseases | digestive | 0.408 | 0.141 | 2.00E-07 | 5487 | 1018 | 4469 |
| 530.11 | GERD | digestive | 0.400 | 0.147 | 3.91E-07 | 5478 | 930 | 4548 |
| 539 | Bariatric surgery | digestive | 0.258 | 0.178 | 2.88E-11 | 5677 | 917 | 4760 |
