# Supplementary material for: Patterns and correlates of mental healthcare utilization during the COVID-19 pandemic among individuals with pre-existing mental disorder
Source: PLoS One. 2024 Jun 4;19(6):e0303079. doi: 10.1371/journal.pone.0303079 (PMC11149861; doi:10.1371/journal.pone.0303079)
Supplement: S7 Table — (DOCX) [file pone.0303079.s010.docx]

| **Phenotype** | **Description** | **Category** | **OR** | **SE** | **p.bonferroni** | **n_total** | **n_cases** | **n_controls** |
| --- | --- | --- | --- | --- | --- | --- | --- | --- |
| 261 | Vitamin deficiency | endocrine/metabolic | 0.393 | 0.166 | 1.68E-05 | 5079 | 790 | 4289 |
| 278 | Overweight, obesity and other hyperalimentation | endocrine/metabolic | 0.400 | 0.130 | 1.83E-09 | 5063 | 1508 | 3555 |
| 278.1 | Obesity | endocrine/metabolic | 0.350 | 0.140 | 5.22E-11 | 5107 | 1346 | 3761 |
| 278.11 | Morbid obesity | endocrine/metabolic | 0.274 | 0.168 | 1.02E-11 | 5158 | 1023 | 4135 |
| 296 | Mood disorders | mental disorders | 2.036 | 0.120 | 2.68E-06 | 4931 | 2465 | 2466 |
| 300.11 | Generalized anxiety disorder | mental disorders | 1.920 | 0.107 | 7.66E-07 | 5104 | 1133 | 3971 |
| 401 | Hypertension | circulatory system | 0.275 | 0.177 | 2.38E-10 | 5112 | 870 | 4242 |
| 401.1 | Essential hypertension | circulatory system | 0.298 | 0.176 | 4.57E-09 | 5116 | 855 | 4261 |
| 530.11 | GERD | digestive | 0.413 | 0.159 | 2.38E-05 | 5025 | 784 | 4241 |
| 539 | Bariatric surgery | digestive | 0.261 | 0.206 | 5.80E-08 | 5176 | 715 | 4461 |
